# Artificial intelligence, health empowerment, and the general practitioner scheme

**DOI:** 10.1177/20552076251365006

**Published:** 2025-07-29

**Authors:** Rune Johan Krumsvik, Marius Ole Johansen, Vegard Slettvoll

**Affiliations:** 1 Department of Education, Faculty of Psychology, 1658 University of Bergen, Bergen, Norway; 2 Faculty of Medicine, 1658 University of Bergen, Bergen, Norway

**Keywords:** Artificial intelligence < general, digital health < general, public health < disease, technology < general, telemedicine < general

## Abstract

The general practitioner (GP) scheme in Norway has long been under pressure, and in recent years, several media outlets have referred to the situation as a “GP crisis.” Over the past year, however, this trend has begun to shift. Bolstered by increased baseline funding, the number of Norwegians without a designated GP has been nearly halved. Predicating this improvement is the fact that GP patient lists are gradually becoming shorter. For the first time, the average GP list now includes fewer than 1000 individuals. To maintain this positive development, either more physicians must choose to become GPs—which would likely come at significant cost to municipalities and the state—or solutions must be implemented that allow for sustainable patient list sizes while mitigating the chronic overload that many GPs have faced. At the same time, artificial intelligence (AI) and large language models may offer promising capabilities in this setting. In this commentary, we explore how AI, wearable devices, everyday technologies, social chatbots, and “health companions” could help alleviate the burden on the GP scheme, empower individuals in managing their own health, and reduce overall societal healthcare costs.

## Introduction

In March 2023, we began pretesting the then-largest language model, GPT-4, and found that it performed remarkably well on one of Norway's most comprehensive medical exams.^1,2^ It also handled two simulated clinical patient cases with promising results.^3^ Since then, a number of individual studies and systematic reviews^4–14^ have reported similar findings, demonstrating that GPT-4 (and later versions), as well as other forms of artificial intelligence (AI), perform well when compared to physicians and radiologists on exams, tests, and clinical case simulations. At the same time, it is important to emphasize that the application of AI in healthcare remains in its early stages, particularly when it comes to large language models. A recent systematic review and meta-analysis, covering primary studies from 2018 to 2024 and drawing on a wide range of language models, presents more mixed results, based on 71 peer-reviewed articles and 12 preprints.^15^

In a previous commentary, we explored how primary healthcare and the general practitioner (GP) system could be supported through rethinking health empowerment, AI, wearables, and everyday technology.^16^ Since GPs are the key “gatekeepers” of primary healthcare, it is essential to examine what can be done to improve conditions for both physicians and patients. In this commentary, we aim to discuss how health empowerment can be strengthened and how the GP system can be supported through the thoughtful use of everyday technologies, wearables, AI, and a “health companion,” while also reducing societal healthcare costs.

## Background

**Figure 1.**
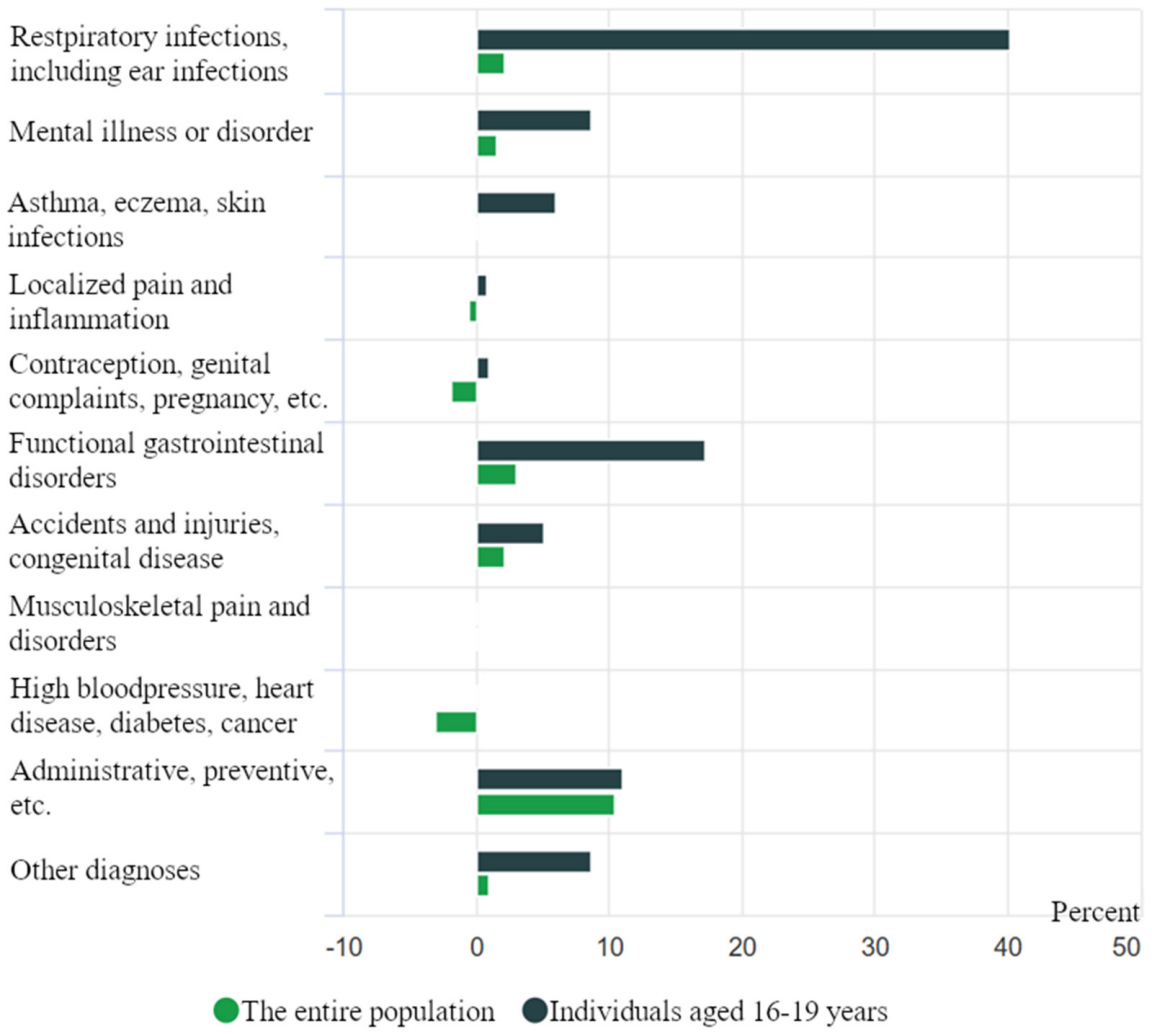
Percentage growth in the number of general practitioner consultations by diagnostic category.^19^

The GP scheme is under significant strain,^17^ and only 3% of medical students and recent graduates in Norway report that they expect to become GPs.^18^ The reasons for this overload are complex, but one illustrative example is the impact of a stricter school attendance policy introduced in upper secondary education a few years ago. This policy added yet another burden for GPs, as it led to a sharp increase in students requesting medical certificates for common ailments such as upper respiratory infections. In fact, the number of consultations per 1000 individuals rose by 40% in the first year after the policy was implemented (see Figure 1), and upper respiratory infections accounted for ∼60% of the increase in doctor visits among young people due to such conditions.^18^

**Figure 2.**
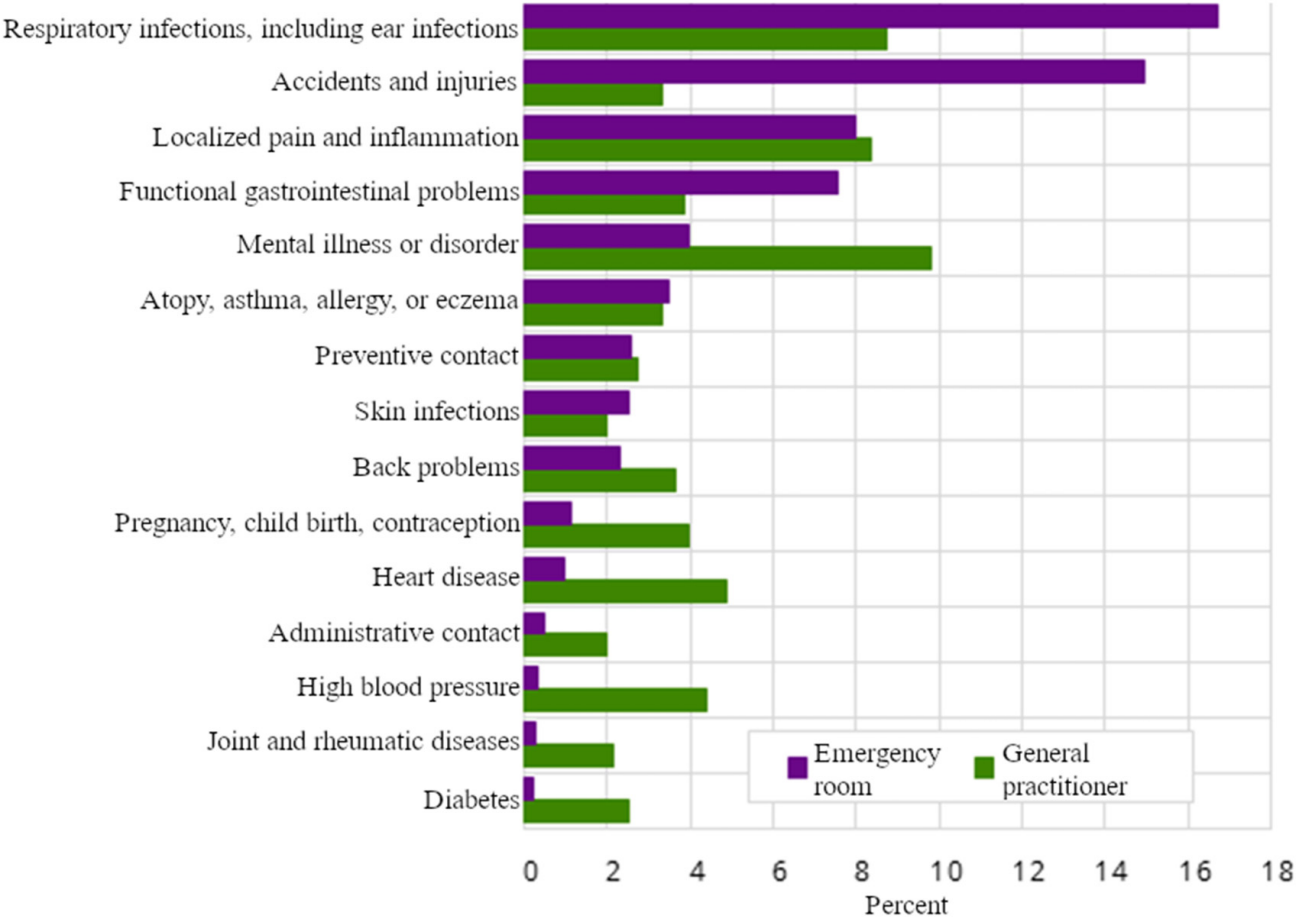
Percentage of consultations with general practitioners and emergency services by selected diagnostic categories.^22^

In retrospect, most have come to view this added strain on the GP system as unnecessary. Both individual studies and international systematic reviews indicate that 30%–60% of visits to GPs in the general population are entirely avoidable.^20,21^ Although the evidence base on this issue in Norway is limited, a few studies from Statistics Norway (Figures 1 and 2) suggest that Norway does not differ significantly from other countries in this regard. The most concerning consequence is that patients who genuinely need to see a GP may be pushed to the back of an increasingly long queue. This disproportionately affects vulnerable patient groups, people living in remote areas, and those who lack the means to access private healthcare services.

## “AI-Supported Self-Help” for the general population?

Several reports on AI in healthcare have been published in recent years,^23^ and in our own AI research at the Digital Learning Communities Artificial Intelligence Centre (DLCAIC^24^),^a^ we have piloted how everyday health technology and AI can contribute to increased health literacy, autonomy, and “health empowerment” among the general population.^25^ Our findings are based on GPT-4's capabilities to analyze clinical patient cases and answer large exams from the final stages of medical school,^1^ GPT-4's performance on the national nursing examination,^26^ feedback at the doctoral level,^27^ methodological capabilities,^28^ trials of wearables,^29^ and the piloting of a self-developed health chatbot.^30^ The research findings suggest that AI, everyday health technology, and wearables, when used in a responsible and well-thought-out manner, have the potential to enhance health empowerment and alleviate some of the pressure on primary healthcare services. However, we lack a solid evidence base in this area, which is why DLCAIC will conduct several studies in Norway in the coming years.

Although we have identified gaps in the existing knowledge, it is worth highlighting that Statistics Norway (SSB) conducted a study in 2015^22^ that mapped the percentage distribution of reasons for visits to GPs (and emergency services).

Figure 2 shows that consultations related to common ailments, such as respiratory infections, account for 8.8% of consultations with GPs, and 16.5% of emergency service visits. However, it is important to note that people visit their GPs ten times more frequently than emergency services, and in 2023, the total number of consultations with GPs was 16.9 million.^31^ Given that the percentage distribution remains largely unchanged, this implies that if just 1% of these consultations could have been avoided through the use of everyday technology, AI, a “health companion,” and wearables, it would strengthen health empowerment and save (relieve) 12.8 full-time positions every year. While this might not seem like a large number at first glance, it is important to note that Norway is a relatively small country with a low population. Even modest improvements in efficiency can have a significant impact on healthcare delivery and resource allocation. This would ensure that patients who truly need GP consultations receive them quickly and effectively. If the rate were increased to 5%, it would correspond to 64.2 full-time positions, and if up to 10% of these consultations could be avoided, it could save 128.3 full-time positions annually.

**Figure 3.**
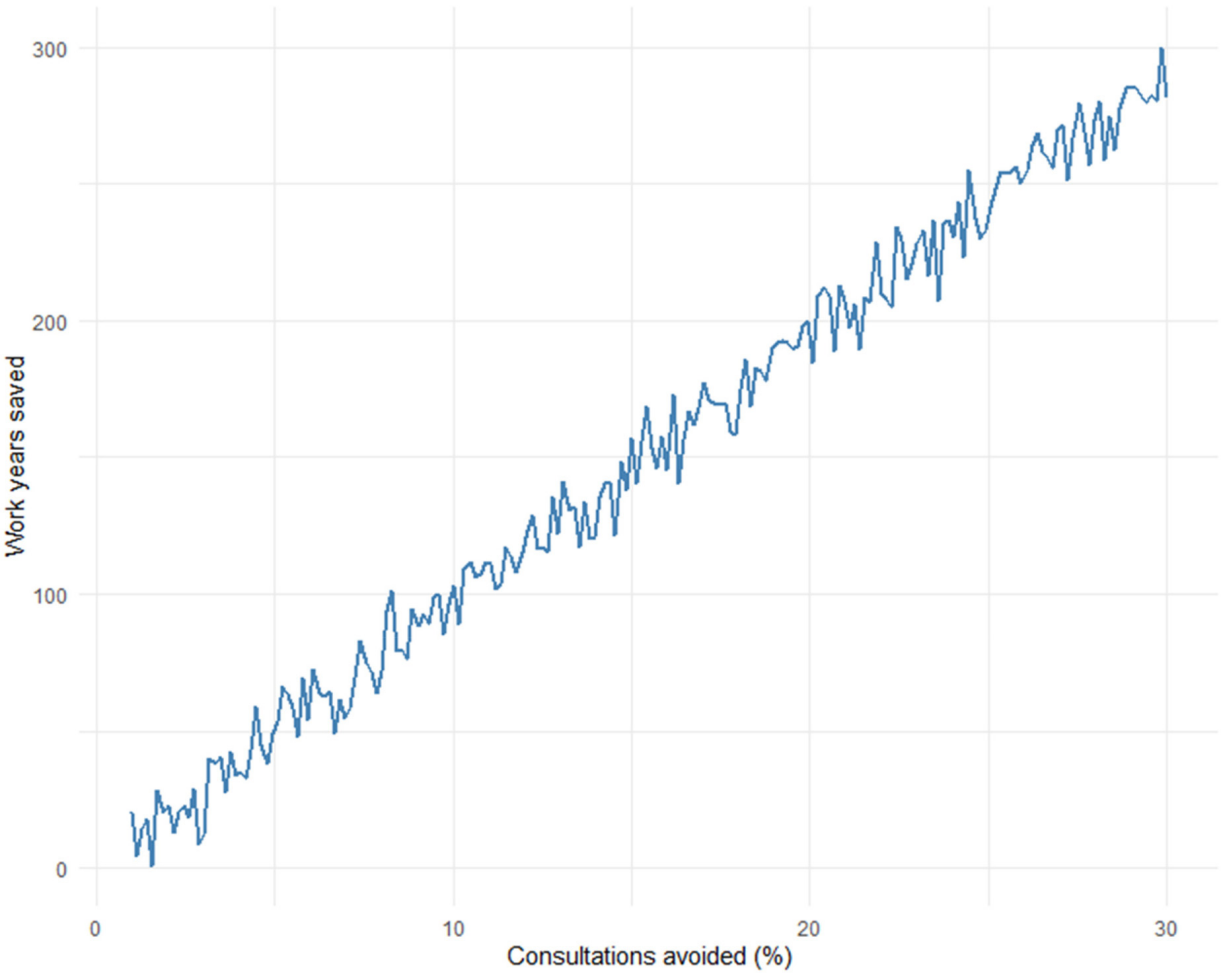
Simulation of how many full-time positions can be relieved in the healthcare sector per year based on the percentage of consultations that could have been avoided.

Based on such simple calculations, Monte Carlo methods could be used to simulate how many full-time positions could be relieved in the healthcare sector each year, based on the percentage of consultations that could have been avoided:

Figure 3 shows the full-time positions saved (or relieved) as a function of the percentage of avoided consultations related to respiratory infections. It is important to note that such models are based on simplified assumptions, like fixed consultation durations, average costs, and full-time equivalence. As such, the figures should be interpreted as exploratory simulations intended to illustrate potential trends rather than as precise predictive estimates. If everyday technology, AI, wearables, and a “health companion” can alleviate 10% of the consultations in this area, it would save the healthcare sector nearly 130 full-time positions every year, while also strengthening health empowerment in the population. So, what exactly is a “health companion”?

In the face of increasing pressure on primary healthcare, an aging population, and the closure of local communities due to extreme weather events, we are beginning to see the emergence of a new and low-threshold actor in the healthcare landscape: the health companion.

The health companion is envisioned as a digital, AI-supported tool, such as an app or interface integrated with wearables and home-based health technologies (e.g. blood pressure monitors, C-reactive protein (CRP) testers, or electrocardiogram (EKG) devices). It leverages AI and large, research-based language models to analyze health indicators, provide early warnings, and offer tailored suggestions or next steps for common ailments. The goal is not to replace professional medical evaluation but to reduce unnecessary GP consultations by supporting individuals in managing minor health concerns independently and responsibly. An element of the health companion concept is the inclusion of a “human in the loop” in the form of a health buddy, which closely resembles the concept of an emergency buddy.^b^ It can be someone in your social circle, extended family, or neighborhood who has a certain level of health knowledge—for example, a nurse, medical student, or Red Cross volunteer—that you can consult after using home-based everyday technologies such as blood pressure monitors, CRP testers, EKG devices, or wearables. In combination with AI and large, research-based language models that provide predictive insights and suggestions based on collected health indicators, such a health buddy could function as a critical “human in the loop” element. A health buddy provides reassurance, evaluation, and human judgment—not to replace the GP, but to help more people manage common health issues in a responsible way without unnecessarily burdening the healthcare system. This could support a form of health empowerment among the general population, which, along with “health hubs”^c^ in remote areas, physical locations in remote or underserved areas where individuals can access AI-enabled tools, basic diagnostics, and low-threshold services. Together, digital health companions, health buddies, and distributed health hubs could relieve the primary healthcare sector, rooted in both AI capabilities and relational trust. But how can we realize such low-threshold measures from an economic standpoint?

Assuming that the employment rate in Norway is 70%,^32^ which corresponds to ∼2.91 million people,^33^ we can draw some interesting conclusions. According to Health Norway, the average consultation with a GP lasts 15 minutes.^34^ However, accounting for waiting time and travel to and from the consultation, we will assume for simplicity that each consultation costs the workplace one hour of absence. According to Statistics Norway (SSB), people typically use an average of three consultations with a GP per year.^35^ This means that these consultations (assuming they occur during working hours) cost society 8.73 million hours every year. This translates to 5150 full-time positions annually. Of course, no one is suggesting that people should not visit their GPs, but the point is that we should avoid everyday complaints from high school students and the general population becoming a burden on GPs, ensuring that those who truly need medical care receive it quickly and professionally.

**Figure 4.**
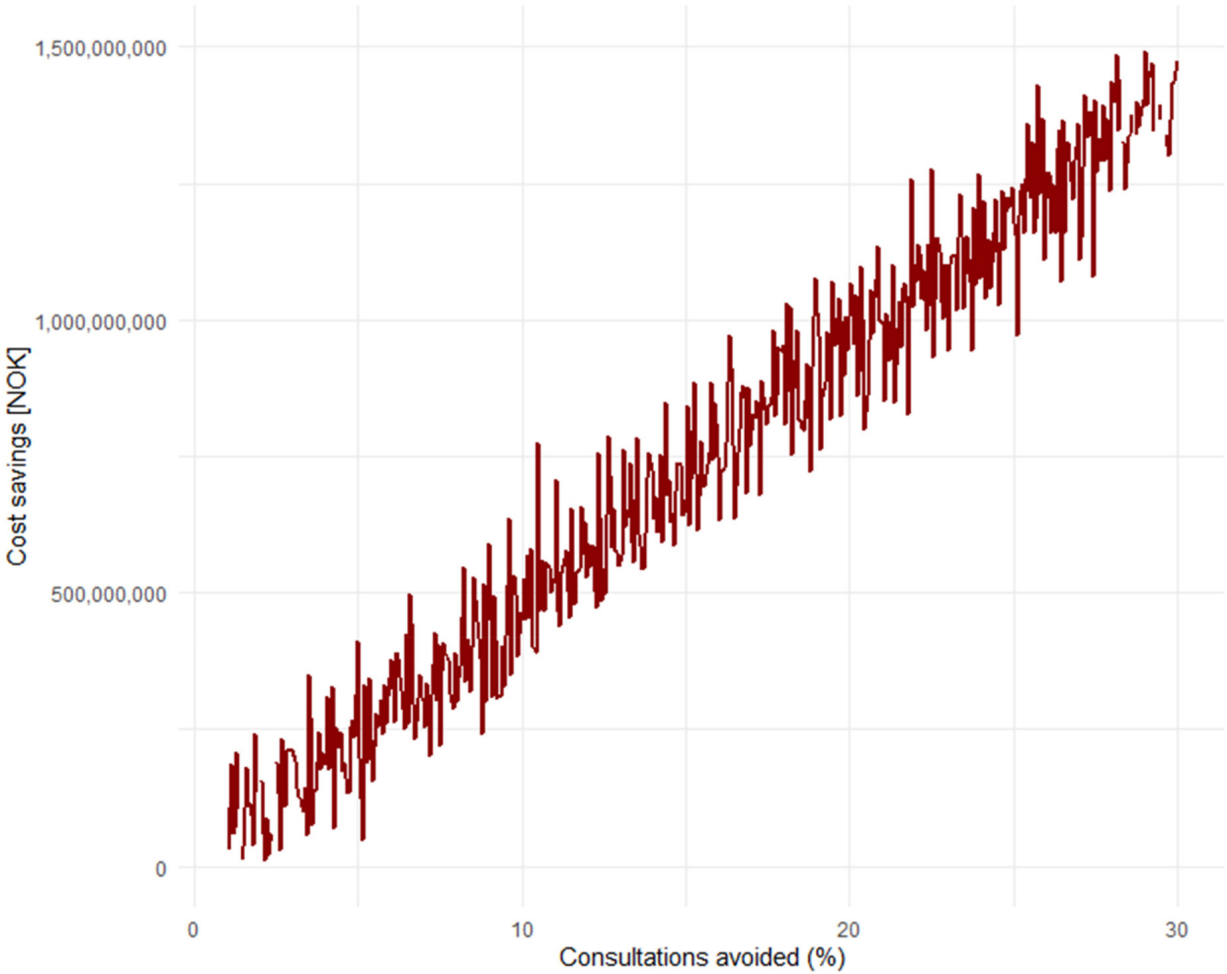
Simulation of annual savings in Norwegian Krone (NOK) as a result of consultations that could have been avoided.

Additionally, given that the average cost of a full-time position in Norway is 923,795 NOK (≈88,700 USD^36^), this amounts to significant societal costs each year. Simple calculations will show that if 10% of these consultations could have been avoided with the use of everyday technology, AI, wearables, and a “health companion,” it would save society a total of 475.8 million NOK (≈45.6 million USD) annually. While there is no concrete data in Norway on the exact percentage of GP visits that could be avoided, this can be considered in light of the aforementioned international studies.^20,21^ If we assume that 30% of consultations could also be avoided in Norway, this would save society an annual amount of 1.4 billion NOK (≈134.4 million USD). Models based on such data can easily be simulated to show the annual savings in terms of avoided consultations (Figure 4).

One could certainly argue that not everyone in employment works full-time, and, therefore, it may be easier for many to schedule a GP appointment outside of working hours. However, it's also worth noting that we haven't included the “cost” of the cognitive burden and health concerns people may experience while waiting for a GP consultation for weeks, which can negatively impact productivity or work ability among the employed. Such forecasts are, in any case, not an exact science (and we don't claim they are), but AI gives us an opportunity to think differently about the workload of GPs, health empowerment, AI, and societal costs.

**Figure 5.**
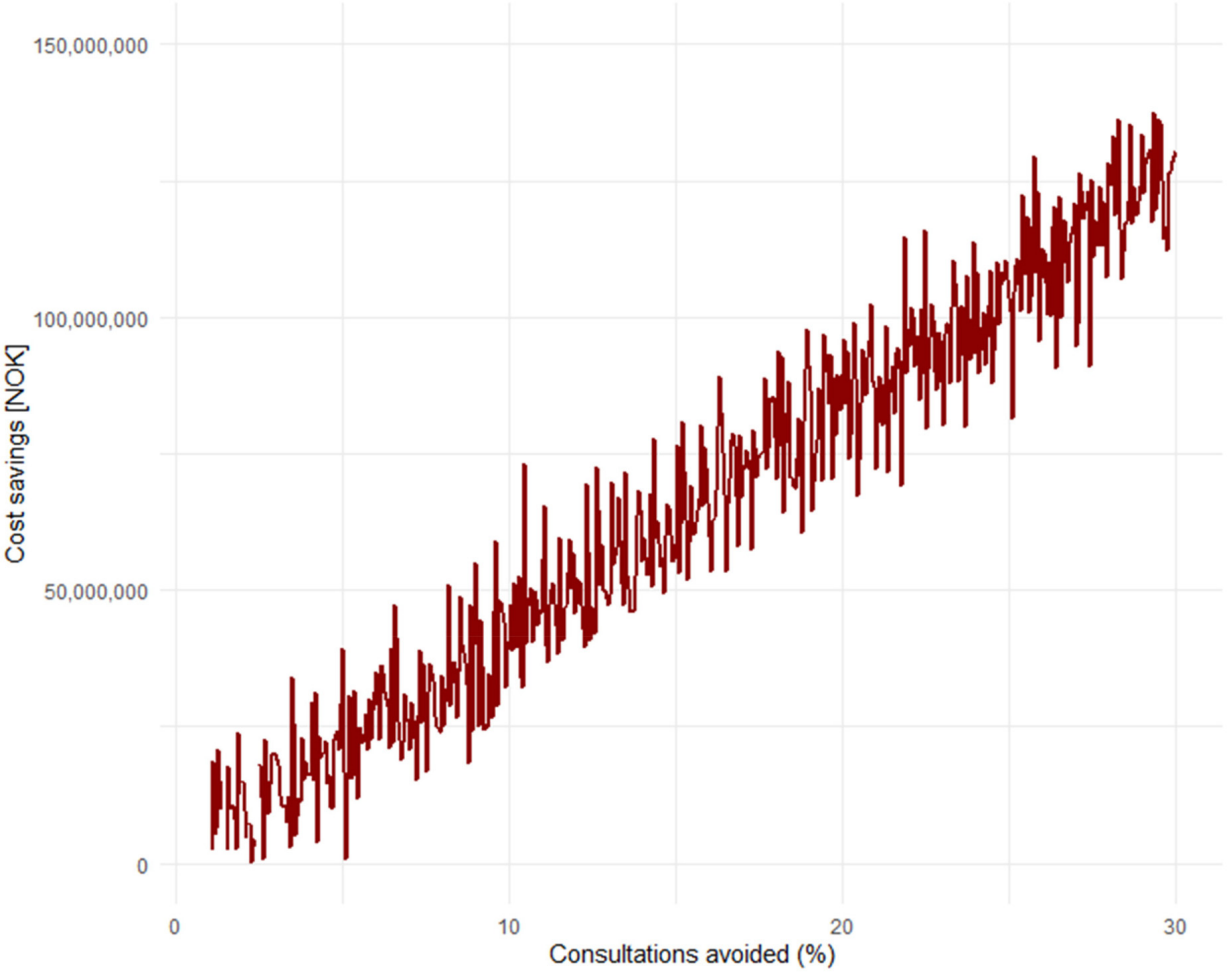
Simulation of how much society can save annually solely from consultations related to respiratory infections.

One can also view this graph (Figure 5) in light of the previous one, by looking at how much society could save annually solely from consultations related to respiratory infections.

It can tentatively be seen that if 5% of consultations related to respiratory infections could have been avoided, society would save an annual amount of 21 million NOK (≈2 million USD), while if up to 30% of such consultations could have been avoided, the annual savings would amount to 126 million NOK (≈12.1 million USD). It is important to note that these projections rest on the assumption that AI-supported tools and wearables can reduce healthcare utilization without compromising care quality. However, the empirical evidence supporting this claim is still emerging, and remains limited for common conditions such as respiratory infections. While early research suggests that AI may assist in triage, risk assessment, and patient education,^4–14^ few large-scale, real-world studies have yet demonstrated consistent reductions in consultation rates with maintained or improved outcomes. Moreover, the distinction between general-purpose language models like GPT-4 and specialized agentic systems trained for diagnostic support or respiratory screening is highly relevant. The latter may offer greater clinical reliability in specific domains, particularly when developed with domain-specific data and validated under clinical supervision. Further research is needed to evaluate how these systems perform in primary care contexts and whether they can reliably support or redirect patients in a safe, efficient, and equitable manner.

The value of removing everyday complaints from the GP's healthcare queues through “AI-assisted self-help” ensures that those who truly need professional help can access it quickly, and this is the most important aspect. At the same time, some of the savings generated could be used to establish low-cost initiatives such as AI-supported “health hubs” in remote areas, strengthening AI-supported health empowerment, and subsidizing robust, research-based AI tools for the general population.

AI-supported health empowerment can also be viewed in light of the fact that in the past few years, several systematic reviews and meta-analyses have been published on the effect of social chatbots on mental (ill)health, depression, health-promoting lifestyle, and more. Zhong et al.^37^ in their systematic review and meta-analysis find that “AI-chatbots demonstrate significant reductions in depression and anxiety.” Li et al.^38^ found that “The meta-analysis revealed that AI-based CAs significantly reduce symptoms of depression and distress. These effects were more pronounced in CAs that are multimodal, generative AI-based, integrated with mobile/instant messaging apps, and targeting clinical/subclinical and elderly populations.” Singh et al.^39^ found that although some of the primary studies had room for improvement, the meta-analysis result showed “significant effects of chatbots for increasing total physical activity, daily steps, fruit and vegetable consumption, sleep duration, and sleep quality.”

While AI models such as GPT-4 have demonstrated strong performance on medical exams,^4–14^ including Norway's comprehensive medical and nursing assessments, it is important to recognize that such results do not directly translate into clinical competence or safe patient care. Exam-based benchmarks provide insight into baseline knowledge but do not account for contextual reasoning, patient rapport, or ethical decision-making. Recent pilot studies and real-world trials have begun to explore the effectiveness of AI in clinical environments. For example, Sorin et al.^40^ analyzed GPT responses to patient questions and found that the chatbots’ advice was rated more empathetic and of higher quality than physician responses. Moreover, recent systematic reviews^41^ emphasize that AI use in primary care is still at an early stage, with promising but mixed results. There remains a clear need for more rigorous clinical evaluations, particularly in diverse, real-world primary care settings.

The findings from these knowledge summaries are particularly interesting because they challenge established notions of how social relationships are formed, maintained, and understood in the context of AI-based interactions. Still, everything suggests that, in the foreseeable future, we will continue to rely mostly on the interpersonal closeness of physical meetings, where the (technology-free) quiet moment still holds the deepest significance. Based on our piloting, pretesting, and publication of the capabilities and reliability of AI at DLCAIC, we see that it is important to address this kind of rethinking and AI innovation to reduce pressure on the primary healthcare system, strengthen health empowerment in the population, and increase access to good everyday healthcare. However, this requires a well-thought-out approach, careful validation, and attention to key ethical concerns, including data privacy, algorithmic bias, and equitable access across different user groups. Yet, broader public acceptance of AI and wearable technologies is not guaranteed. Factors such as trust in automated decision-making, cultural attitudes, and varying levels of comfort with technology will likely influence real-world adoption, regardless of technical capability. While AI tools and wearables hold great promises for supporting health empowerment and alleviating pressures on primary healthcare, their real-world use involves several important limitations and risks that must be acknowledged. First, false reassurance is a significant concern.^42,43^ If AI-based assessments incorrectly indicate that a condition is benign or manageable, patients may delay seeking necessary medical care. Conversely, overdiagnosis can lead to unnecessary anxiety, additional testing, and overtreatment.^44^ Further, alert fatigue is a known issue, where frequent notifications and warnings from devices may desensitize users, causing them to ignore or override important alerts.^45^ This can undermine the effectiveness of monitoring and reduce patient safety. Third, technical failures and inaccuracies can arise from sensor malfunctions, data transmission errors, or algorithmic biases embedded in AI models.^46^ Such issues might disproportionately affect certain populations, raising concerns about equitable access and the risk of exacerbating health disparities. Finally, effective implementation depends not only on technology performance but also on appropriate user training, digital literacy, and continuous oversight by healthcare professionals to ensure safe and beneficial integration.

The health companion is introduced here as a conceptual framework rather than a fully defined role. Its implementation would require careful consideration of several practical and regulatory issues, including training requirements, legal liability, continuity of care, and compensation if human actors are involved. Likewise, if patients rely on AI-supported systems, clear protocols must be in place for handling technological failures, user-training, and assigning responsibility. These issues merit further exploration before any large-scale adoption and should be guided by evidence, ethics, and patient safety.

## Notes

a. This is particularly addressed in Work Package 1 and Work Package 5, where we build upon our previous DLC research in health-related areas, as well as the entire DLCAIC consortium's research.

b. Crisis companion: https://naob.no/ordbok/beredskapsvenn.

c. For example, more rural and local variants of such “Emergency Friends” could be translated as “health buddies,” “rural or community-based crisis companions” or “local emergency support networks”: HelseHub – Norway Health Tech and Helsehub.
